# Use of Biostimulants: Towards Sustainable Approach to Enhance Durum Wheat Performances

**DOI:** 10.3390/plants11010133

**Published:** 2022-01-04

**Authors:** Sourour Ayed, Imen Bouhaouel, Hayet Jebari, Walid Hamada

**Affiliations:** 1 Field Crops Laboratory, LR20-INRAT-02, National Agricultural Research Institute of Tunisia, University of Carthage, Ariana 2049, Tunisia; hayetjebari99@gmail.com; 2 Genetics and Cereal Breeding Laboratory, LR14AGR01, National Agronomic Institute of Tunisia, University of Carthage, Tunis 1082, Tunisia; imenbouhaouel@gmail.com (I.B.); w_hamada@yahoo.com (W.H.)

**Keywords:** *Triticum durum*, *Bacillus* spp., *Trichoderma* spp., *endomycorrhiza*, seed coating, foliar spray, sustainability, semi-arid environment

## Abstract

The use of biostimulant (BS) holds a promising and environmental-friendly innovation to address current needs of sustainable agriculture. The aim of the present study is twofold: (i) assess the potential of durum wheat seed coating with microbial BS (‘Panoramix’, Koppert), a mix of *Bacillus* spp., *Trichoderma* spp., and *endomycorrhiza,* compared to two chemical products (‘Spectro’ and ‘Mycoseeds’) through germination bioassay, pot and field trials under semi-arid conditions, and (ii) identify the most effective method of BS supply (‘seed coating’, ‘foliar spray’, and ‘seed coating + foliar spray’) under field conditions. For this purpose, three modern durum wheat cultivars were tested. ‘Panoramix’ was the most efficient treatment and enhanced all germination (germination rate, and coleoptile and radicle length), physiological (relative water content, chlorophyll content, and leaf area), and agro-morphological (plant height, biomass, seed number per spike, thousand kernel weight, and grain yield) attributes. Unexpectedly, the individual application of ‘Panoramix’ showed better performance than the combined treatment ‘Panoramix + Spectro’. Considering the physiological and agro-morphological traits, the combined method ‘seed coating + foliar spray’ displayed the best results. Principal component analysis confirmed the superiority of ‘Panoramix’ treatment or ‘seed coating + foliar spray’ method. Among tested durum wheat cultivars, ‘Salim’ performed better especially under ‘Panoramix’ treatment, but in some case ‘Karim’ valorized better this BS showing the highest increase rates. Based on these study outcomes, ‘Panoramix’ might be used as promising sustainable approach to stimulate durum wheat performance.

## 1. Introduction

Various agricultural approaches can be used within an integrated farming system to increase grain yield as well as quality, and alleviate stress-induced limitations. Recently, the use of agricultural biostimulants (BSs) has emerged as a valid alternative to agrochemicals and to indirectly sustain plant growth and productivity [1]. BS was defined as products of biological origin and included many products that are described as metabolic enhancers, phyto-stimulators, biofertilisers, biogenic stimulants, plant growth regulators, plant strengtheners, biocontrol agents, elicitors, allelopathic preparations, and plant conditioners [2]. In general, a plant BS is any natural substance applied to soil/plant that can be classified into several categories: (i) humic substances, (ii) complex organic materials (obtained from agro-industrial and urban waste products, sewage sludge extracts, composts, and manure), (iii) beneficial chemical elements (e.g., aluminium [Al], cobalt [Co], sodium [Na], selenium [Se], and silicon [Si]), and (iv) inorganic salts including phosphite (HPO_3_^2−^), seaweed extracts [3]. In addition, plant BS might include beneficial microorganisms such as mycorrhizal and non-mycorrhizal fungi, bacterial endosymbionts (e.g., *Rhizobium*), and plant growth-promoting rhizobacteria (PGPR).

Among microbial BSs, *Bacillus* and *Pseudomonas* species are the predominant PGPR widely used as biofertilizers [4]. However, *Bacillus*-based bio-fertilizers are more active compared to *Pseudomonas*-based fertilizers [5]. This has been attributed to the production of more effective metabolites and spore forming character of *Bacillus* spp., which increases the viability of cells in commercially formulated products. The plant-beneficial *Bacillus* spp. associate with roots or rhizosphere and develop biofilms, allow the assembly of cells embedded in a matrix composed of exopolysaccharides and proteins which indirectly protect plants by inducing systemic resistance and thus increase plant growth and final yield [6].

Some BSs might contain beneficial fungi such as *Trichoderma* spp., common free-living fungi in the rhizosphere and soil [7]. *Trichoderma* spp. is considered as the most important filamentous fungi in biological control strategies [8,9] and an excellent plant growth-promoting fungi (PGPF) [10,11]. *Trichoderma* spp. is present as an active ingredient in more than 200 products worldwide, e.g., biopesticides, biofertilizers, bio-growth enhancers. Moreover, it is marketed for conventional and organic agricultural production [12,13]. *Trichoderma*-based products are known for their potential to improve plant growth, productivity, nutritional quality as well as resistance to plant pathogens/pests and numerous environmental stresses [8,11,12].

BSs might include also arbuscular mycorrhizal (AM) fungi, the so-called *endomycorrhiza*, which are obligate symbiotic fungi that predominate roots and rhizosphere [14]. In this symbiotic relationship, the crops supply lipids and/or sugars to their symbionts, thus providing the fungi with a source of carbon for their metabolic needs [15]; in return, the fungi provide benefits to their associated hosts [16]. Several AM fungal species, e.g., *Glomus intraradices*, *Glomus mosseae* (renamed to *Funneliformis mosseae*), *Rhizophagus irregularis*, and *Rhizophagus fasciculatus*, have been used to improve crop performance [17].

Currently, some commercial BS products combine the effects of several beneficial microorganisms such as *Bacillus* spp., *Trichoderma* spp., and AM fungi that might be applied according to several methods. BSs are usually added to the plant by seed priming, seed coating, foliar spray or root dipping and/or to the soil by direct soil application [17]. Among these methods, seed coating is one of the most promising techniques for delivering beneficial microbes, as it allows small amounts of inocula to be used in a specific application. Inspired from the pharmaceutical industry, seed coating was first applied to cereal seeds in the 1930s, and thereafter, its large-scale commercial use began in the 1960s [18]. Seed coating is the application of exogenous materials onto the surface of seeds in order to improve their appearance and handling characteristics (e.g., seed shape, weight and size). It can also be used to deliver active compounds (e.g., plant growth regulators, micronutrients, and microbial inoculants) that stimulate healthy and rapid establishment, and thereby maximize crop production [19,20]. Otherwise, the literature reports several positive effects of BS foliar spray on plant growth, productivity, and quality [21,22]. BS foliar spray is largely used, but this method is expensive and requires large amounts of inoculant [17].

Most of these BS application methods have shown a beneficial effect on crop yield by (i) stimulating germination, vegetative and reproductive growth especially the root development (density and depth), and (ii) improving plant resilience to biotic/abiotic stress after induction of plant biological activity [23,24,25]. In fact, BSs enhance the crop’s physiological and biochemical processes by regulating hormones balance, enhancing photosynthetic activity, nutriment uptake, and nutritional efficiency [12,13,26]. Furthermore, BSs could improve the physicochemical properties of soil, the activity of rhizosphere microbes and soil enzymes, and therefore the nutrient bioavailability [1]. Although BSs are beneficial for crop production systems, their modes of action remain largely unknown [27,28]. This might be due in part to the great diversity of raw materials (e.g., strain of microorganisms) used in the manufacture of each product, in addition to the diversity related to species or genotypes. In this sense, there is little documented literature on the effects of exogenous BSs on durum wheat (*Triticum durum* Desf.) production. In Tunisia, this species constitutes the largest part of the staple food. However, enhancing sustainable productivity is facing concomitant challenges, especially in semi-arid environment [29,30]. In this context, the present study aims to assess (i) the effect of a BS, a microbial consortium of *Bacillus* spp., *Trichoderma* spp., and AM fungi, on germination, physiological and agro-morphological attributes under different experimental conditions (Petri dish, pot, and field trials), (ii) the effectiveness of three supply methods of BS under field conditions, and (iii) the genotypic response of three modern durum wheat cultivars to BS treatment and supply methods.

## 2. Results

### 2.1. Effects of Seed Coating Treatments on Germination Attributes

**Figure 1:**
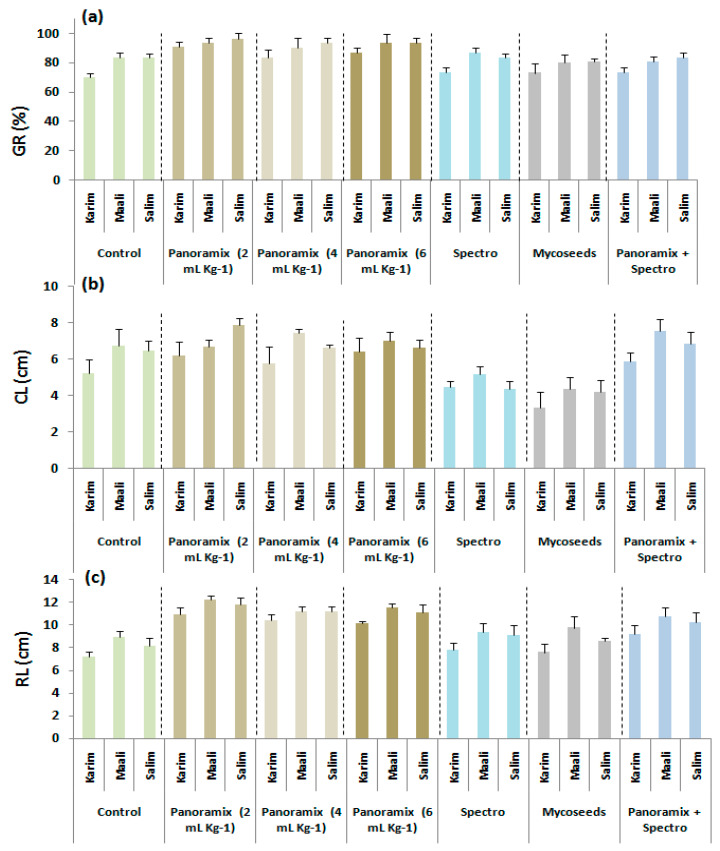
Effects of seed coating treatments on germination rate (GR, **a**), coleoptile (CL, **b**), and radicle (RL, **c**) length of durum wheat cultivars.

The analysis of variance revealed significant differences (*p* ≤ 0.05 and *p* ≤ 0.001) among seed coating treatments and cultivars for all germination attributes (Table 1). Double interaction ‘treatment × cultivar’ was only significant for radicle length (*p* ≤ 0.05). Overall, the results showed that seed coating with BS or chemical products improved germination attributes, except ‘Mycoseeds’ for germination rate, and ‘Spectro’ and ‘Mycoseeds’ for coleoptile length (Table 1, Figure 1). Interestingly, the highest germination rate, coleoptile length, and radicle length were recorded with ‘Panoramix’ using 2 mL kg*^−^*^1^ seeds, inducing respectively an increase rate of 15, 11, and 30% compared to the control. This treatment was followed by ‘Panoramix’ using 6 mL kg*^−^*^1^ (13, 8, and 26%) and 4 mL kg*^−^*^1^ (11, 7, and 26%). A differential mode of action of the used products was observed; i.e., ‘Panoramix’ improved root development more than that of coleoptile, and vice versa for the chemical treatments (‘Spectro’ and ‘Mycosseds’).

During this experiment, a genotypic variation was obtained (Table 1, Figure 1). For the seven seed treatments, ‘Salim’ performed better for the germination rate, while ‘Maali’ exhibited the best values for the coleoptile length and radicle length. Nonetheless, ‘Salim’ showed the highest coleoptile length using 2 mL kg*^−^*^1^ of ‘Panoramix’. It is worth noting that ‘Karim’ displayed the highest increase rates for the germination rate and coleoptile length using ‘Panoramix’ (2, 4, and 6 mL kg*^−^*^1^), but its values still lower than those of ‘Maali’ and ‘Salim’.

**Figure 2:**
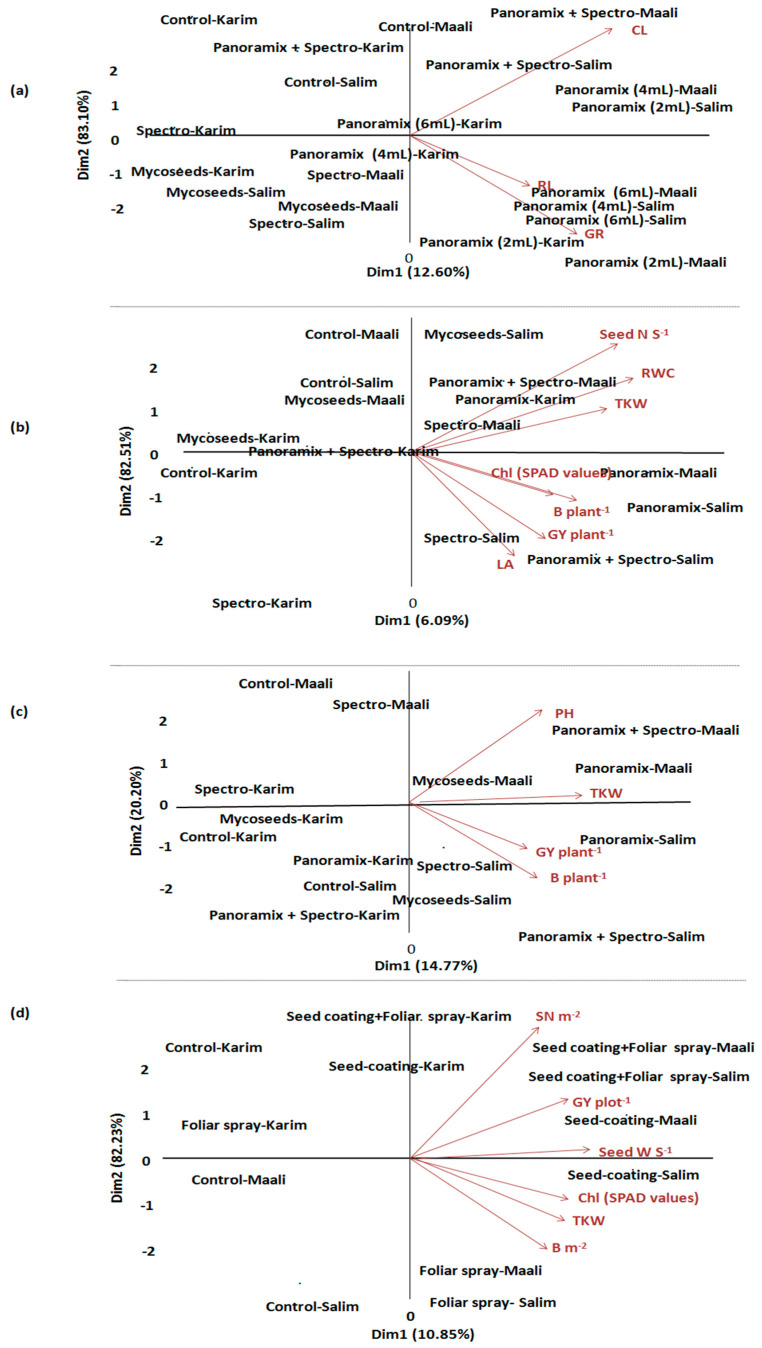
Two-dimensional principal component analysis (PCA) of all combinations (treatment-cultivar) for the germination (**a**), pot (**b**) and field (**c**,**d**) trials.

The PCA was applied in order to evaluate the relation between cultivars and applied treatments. The first and the second principal components (PC-1 and PC-2) accounted for 83 and 13%, respectively, of the total data variance; i.e., their mutual projections (Figure 2, Table S1). Three groups might be discerned: the first group combined the chemical treatments (‘Spectro’ and ‘Mycoseeds’), the second group was mainly constituted by all ‘Panoramix’ treatments, while the last group included the combined treatment, ‘Panoramix + Spectro’. The PCA results confirmed the superiority of ‘Panoramix’ treatment, in particular using the 2 mL kg*^−^*^1^ concentration. The BS enhanced mainly the germination rate and radicle length, but its effect depends on the interaction between concentration and cultivar.

### 2.2. Effects of Seed Coating Treatments on Physiological and Agro-Morphological Attributes of Durum Wheat Cultivars Grown under Pot and Field Conditions

**Figure 3:**
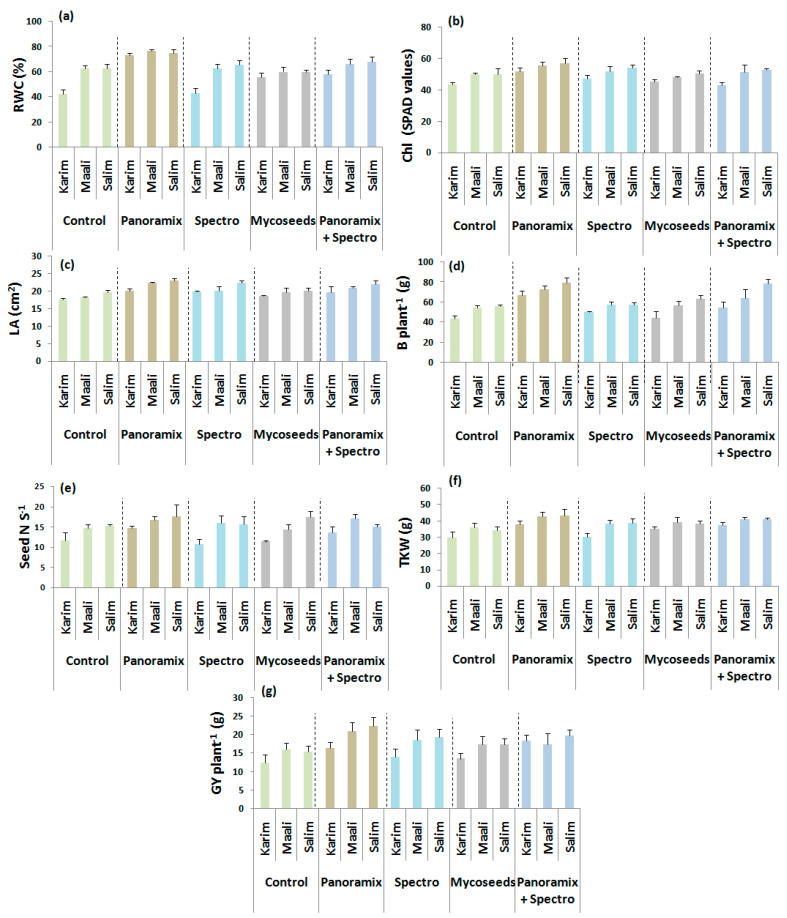
Effects of seed coating treatments on relative water content (RWC, **a**), chlorophyll content (Chl, **b**), leaf area (LA, **c**), biomass (B plant^−1^, **d**), seed number per spike (Seed N S^−1^, **e**), thousand kernel weight (TKW, **f**), and grain yield per plant (GY plant^−1^, **g**) of durum wheat cultivars grown under pot conditions.

For pot trial, statistical differences (*p* ≤ 0.05, *p* ≤ 0.01, and *p* ≤ 0.001) were obtained among seed coating treatments and cultivars for the relative water content, chlorophyll content, leaf area, biomass, seed number per spike, thousand kernel weight, and grain yield, except the insignificant effect for plant height (Table 2). A significant interaction between treatments and cultivars was noted for almost all traits (*p* ≤ 0.05 and *p* ≤ 0.01), except the relative water content, thousand kernel weight, and grain yield. The application of BS or chemical products to the seeds showed a promising effect on physiological and agro-morphological attributes, while no effect was depicted for the plant height (Table 2, Figure 3). As expected, ‘Panoramix’ was the most effective seed treatment and enhanced the relative water content (25%), chlorophyll content (13%), leaf area (14%), biomass (30%), thousand kernel weight (19%), and grain yield (26%) compared to the control. This treatment was followed by the combination ‘Panoramix + Spectro’. ‘Panoramix’ and ‘Panoramix + Spectro’ have more enhanced the biomass and grain yield. Unexpectedly, ‘Spectro’ (relative water content, plant height, seed number per spike, and thousand kernel weight) and ‘Mycoseeds’ (chlorophyll content, leaf area, biomass, and grain yield) have slightly improved some traits.

The three cultivars responded differently to the seed coating treatments (Table 2, Figure 3). Regardless treatments, ‘Maali’ followed by ‘Salim’ showed the highest values for most studied traits. Notably, ‘Salim’ showed the best performance under ‘Panoramix’ treatment, but the best increase rates for the relative water content, chlorophyll content, biomass, and seed number per spike were obtained for ‘Karim’.

The distribution of ‘treatment-cultivar’ combinations was performed on the main plan of the PCA where the first two axes (PC-1 and PC-2) presented 83% of the total variability (Figure 2, Table S2). Based on the identified groups, the results confirmed the noteworthy effect of ‘Panoramix’ as treatment. This BS seems to act on the similar way on ‘Maali’ and ‘Salim’ by enhancing mainly biomass and grain yield.

**Figure 4:**
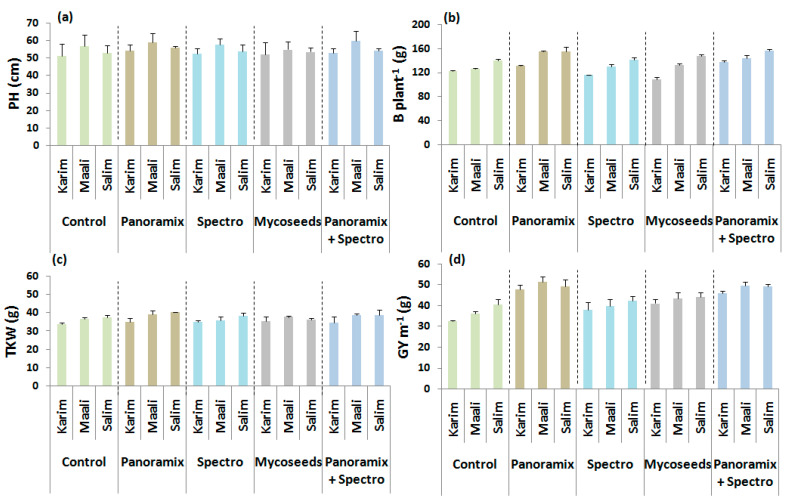
Effects of seed coating treatments on plant height (PH, **a**), biomass per plant (B plant^−1^, **b**), thousand kernel weight (TKW, **c**), and grain yield per linear meter (GY m^−1^, **d**) of durum wheat cultivars grown under field conditions.

For field trial, obtained data showed significant differences (*p* ≤ 0.05 and *p* ≤ 0.001) between seed coating treatments for all agro-morphological parameters, except the seed number per spike (Table 3). However, differences between cultivars were only observed for biomass, seed number per spike, and grain yield (*p* ≤ 0.05 and *p* ≤ 0.01). The double interaction between treatments and cultivars was significant (*p* ≤ 0.001) for almost all traits, except the plant height. As shown in pot trial, ‘Panoramix’ and ‘Panoramix + Spectro’ showed the highest increase rates for the plant height (5 and 5%), biomass (12 and 11%), thousand kernel weight (5 and 4%), and grain yield (27 and 25%) (Table 3, Figure 4). The grain yield was the best improved trait by these two treatments. However, ‘Spectro’ and ‘Mycoseeds’ showed a poorer performance for the plant height, biomass, and seed number per spike. Under semi-arid environment, ‘Salim’ followed by ‘Maali’ responded better to seed coating treatments. Nonetheless, under ‘Panoramix’ treatment, the highest increase rates for grain yield and plant height were observed for ‘Karim’.

Based on PCA results, the PC-1 and PC-2 presented 87% of the total variability (Figure 2, Table S3). This analysis showed that the three durum wheat cultivars acted to ‘Panoramix’, chemicals and combined treatment. This made it possible to group these treatments into the same group for each cultivar. The superiority of ‘Panoramix’ is clearly visible within each group.

### 2.3. Effectiveness of Biostimulant Supply Methods

**Figure 5:**
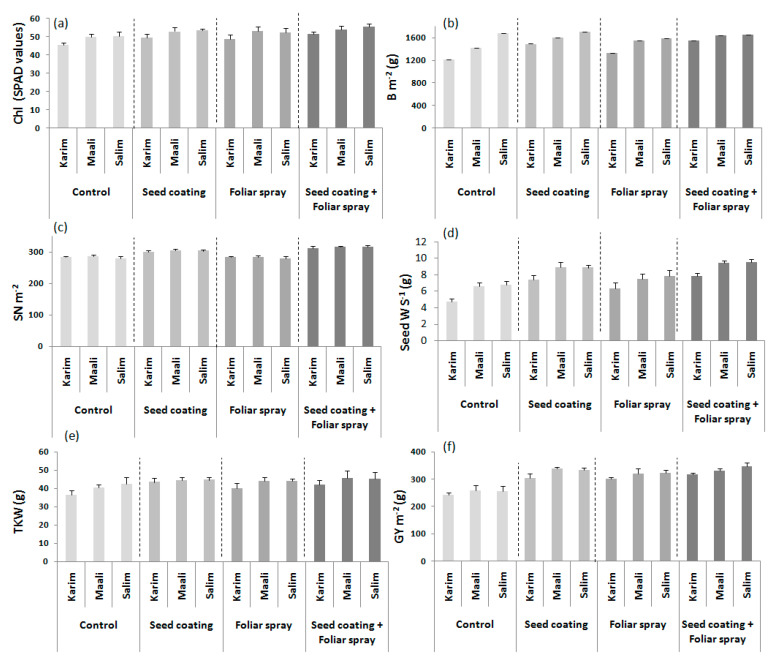
Effects of biostimulant supply methods on chlorophyll content (Chl, **a**), biomass per m^−2^ (B m^−2^, **b**), spike number per m^−2^ (SN m^−2^, **c**), seed weight per spike (Seed W S^−1^, **d**), thousand kernel weight (TKW, **e**), and grain yield per m^2^ (GY m^−2^, **f**) of durum wheat cultivars grown under field conditions.

The results revealed significant (*p* ≤ 0.05, *p* ≤ 0.01, and *p* ≤ 0.001) effect of the applied BS methods on chlorophyll content, biomass, seed weight per spike, spike number, thousand kernel weight, and grain yield (Table 4). Differences between cultivars were noted for the biomass, spikelet number per spike, seed weight per spike, thousand kernel weight, and grain yield. In addition, significant (*p* ≤ 0.05 and *p* ≤ 0.001) interactions between the main factors were observed for biomass and grain yield. Under semi-arid conditions, the application of BS with various methods increased most traits compared to the control (Table 4, Figure 5). Interestingly, the combined methods ‘seed coating + foliar spray’ enhanced the chlorophyll content, biomass, spike number, seed weight per spike, thousand kernel weight, and grain yield by 54, 11, 10, 32, 10, and 24%, respectively. This treatment was followed by the ‘seed coating’ method. Globally, seed weight per spike followed by grain yield were the most enhanced traits by the three methods. Considering the tested durum wheat cultivars, ‘Salim’ was the best performing genotype in most cases.

For the PCA, PC1 and PC2 accounted for 93% of the total variability (Figure 2, Table S4). This analysis showed on one hand that the responses of ‘Salim’ and ‘Maali’ were very close using the three techniques (‘seed coating’, ‘foliar spray’ and ‘seed coating + foliar spray’), and on other hand the high efficiency of the combined method, in particular for ‘Salim’ and ‘Maali’.

## 3. Discussion

### 3.1. Promising Effect of the Biostimulant ‘Panoramix’ on Durum Wheat Germination, Physiological, and Agro-Morphological Performances

Under different experimental conditions, the use of the BS ‘Panoramix’ showed a promising effect on germination, physiological, and agro-morphological attributes compared to the control and chemical products. Therefore, these findings highlight the beneficial effect of the BS, containing a mix of eukaryotic and prokaryotic microorganisms, on plants.

The seed coating could be an effective approach to help plants in germination process. In the present research, the BS ‘Panoramix’ was the most efficient treatment for the germination attributes (i.e., germination rate, coleoptile length, and root length), in particular using 2 mL kg^−1^. These results might be explained by the additive and/or synergetic effect of *Bacillus* spp., *Trichoderma* spp., and AM fungi. In fact, seed coating with saprophytic fungi such as *Trichoderma harzianum*, enhanced the germination rate, shoot and root length, and seed vigor of ‘Karim’ cultivar [31]. In addition, these authors noted an increase in the total phenolic content and peroxidase activity in leaves. More specifically, inoculation with *T. harzianum* enhanced phase III imbibitions; i.e., cell elongation followed by radicle protrusion [32]. The promotive effect of *Trichoderma* on wheat seed germination was attributed to increasing gibberellic acid (GA3) that boosts up the activity of hydrolytic and proteolytic enzymes acting to mobilize the food reserves from the cotyledons or endosperm [33]. Otherwise, *Bacillus* spp. as with *Bacillus subtilis* QM3 increased the isoenzyme activity, *β*-amylase that may be one of the key factors to promote the germination of wheat seeds [34]. The same strain enhanced the antioxidant activities of wheat seeds under salt stress [35].

Unlike chemicals, ‘Panoramix’ enhanced the radicle growth more than the coleoptile growth. This result was probably due to the fact that, when the *Trichoderma* hyphae colonizes and establishes a close relationship with roots, this fungus increases the specific area and robustness of this organ [32,36]. In fact, *T. harzianum* found along the root surfaces and underneath the outermost layer of root cells, released auxin, harzianolide, and harzianic acid [37]. These secondary metabolites promote better the root development (root length and root tips) [38,39,40] allowing to explore a bigger region of soil [26]. Subsequently, the proper development of a root system during germination, as observed in this study, should influence the ability of a good seedling establishment and the further course of seedling growth.

Under semi-arid conditions, ‘Panoramix’ (2 mL kg^-1^) still the best seed coating treatment for durum wheat cultivars. Notably, the individual application of ‘Panoramix’ performs better than the combined application with ‘Spectro’. The positive role of the BS was noted for the three physiological parameters including the relative water content, the chlorophyll content, and the leaf area. In addition, a beneficial effect of the BS on biomass, grain yield and its related components (i.e., seed number per spike and thousand kernel weight) was observed. The induced mechanisms by *Bacillus*, *Trichoderma* or AM fungi might explain the improvement of physiological activity, growth, and yield. In fact, *Bacillus* spp., one of the predominant PGPRs, are known to be associated with roots or rhizosphere and develop biofilms that trigger plant growth and prevent pathogen infection [6,41]. *Bacillus* spp. manipulate the intracellular phytohormone metabolism by the synthesis of indole-3-acetic acid (IAA), gibberellic acid, cytokinins, and 1-aminocyclopropane-1-carboxylate (ACC) deaminase [42,43,44,45]. In particular, IAA has important effects on root growth [46] and architecture [47], while the secretion of ACC deaminase inhibits ethylene synthesis in crop plants and promotes root as well as shoot cell division and elongation [44,48]. In addition, *Bacillus* spp. produce exopolysaccharides and siderophores, which prevent the movement of toxic ions, adjust the ionic balance, water transport in plant tissues and the activation of the antioxidant and defense systems [41]. Some *Bacillus* spp. enhance the nutrient uptake and content in plant tissues [49,50,51,52,53]. Otherwise, the success of *Bacillus* spp. is due in part to its ability to modify the rhizosphere. The hasten growth of plant induced by *Bacillus* spp. might increase the abundance and the activity of this microbial population through the secretion of significant amounts of root exudates [54]. This will in turn increase the availability of nutrients for the microbial consumption. Enhanced microbial activity will have therefore a positive effect on nutrient bioavailability.

Similar to *Bacillus* spp., *Trichoderma* spp. are well known for their ability to produce a wide range of plant-growth promoting substances (secondary metabolites, phytohormones [e.g., IAA and their analogous], vitamins, and enzymes) [55]. These *Trichoderma*-induced mechanisms can influence several aspects of plant development including plant growth and root architecture (increase in the length of lateral and primary root), and nutritional status (increase in nutrient uptake and use efficiency) [11,12,17,55]. As shown for *Bacillus* spp., applying *Trichoderma* spp. to the soil stimulates the root exudation to promote both microbial and plant growth [56]. Moreover, *Trichoderma* spp. produce organic acids in rhizosphere such as gluconic, citric, and/or fumaric acids that decrease soil pH [7].

Otherwise, *endomycorrhiza*, one of the components of ‘Panoramix’ product, has also been shown to improve productivity of numerous crop plants [16], including wheat [57]. In this sense, Zhang et al. [58] reported that inoculation of AM fungi in field increased grain yield by 16%. This symbiotic fungus promotes plant growth by producing metabolites and increasing the acquisition of immobile nutrients such as phosphorus, zinc, and copper beyond the range of plant roots via their hyphae [16,17]. Moreover, other factors associated with AM fungi colonization may influence plant resistance to drought. These factors include changes in leaf elasticity, improving leaf water and turgor potentials, maintaining the stomatal opening and transpiration, increasing root length and depth, and adventice root formation [59,60]. According to Adesemoye et al. [61], the combined inoculation of a strain of AM fungi with two PGPR strains (*Bacillus amyloliquefaciens* and *Bacillus pumilus*), used as a complementary mineral fertilization, can reduce the use of conventional fertilizer by 25%.

### 3.2. Genotypic Variation against Seed and Foliar Treatments

For the germination, pot, and field trials, meaningful and consistent differences between durum wheat cultivars were observed in their response to seed coating treatments or BS supply methods. At germination stage and regardless the seed treatments, ‘Salim’ showed the best germination rate, while ‘Maali’ displayed the highest coleoptile and root length. Under pot conditions, ‘Maali’ exhibited also the best values for physiological and agro-morphological traits, but ‘Salim’ outperformed the others genotypes using ‘Panoramix’. ‘Salim’ was also the best genotype under field conditions for most treatments including ‘Panoramix’ and responded better to the three methods of BS application. This variation might be explained by the genetic variability of the plant species whose seeds were treated by the mix of *Bacillus* spp., *Trichoderma* spp., and AM fungi, as well as their interaction that might influence the action mechanisms of microorganisms. In summary, the best performance was obtained for ‘Salim’ with ‘Panoramix’, but it was not the only genotype that best valorized this BS. Indeed, for some parameters, ‘Karim’ showed the highest increase rates.

### 3.3. Effectiveness of Biostimulant Depends on the Supply Methods

In most cases, the three BS supply methods enhanced the physiological and agro-morphological attributes. The present investigation showed also differences among methods of BS application. The combined technique, ‘seed coating + foliar spray’ was found more effective than the individual applications. The results corroborate with those of Norrie and Keathley [62], Gajc-Wolska et al. [63], and Sharma et al. [64] who depicted that spray application and seed priming boost photosynthetic activity and increase yield of several species. Otherwise, the individual effect of BS using the ‘seed coating’ technique showed a better performance compared to the ‘foliar spray’, contrary to Amutha et al. [65]. Indeed, seed coating, a process that consists in covering seeds with low amounts of exogenous materials, is gaining attention as an efficient delivery system for beneficial microoragnisms [17]. The coating method facilitates the contact between the treatment and the seed [66,67]. This will help beneficial microorganisms to successfully colonize the roots to obtain subsequently healthy, homogeneous and robust seedlings with better nutrients uptake, which constitutes the solid foundation for high yield potential. The colonization of roots at early germination stage might explain the advantageous effect of ‘seed coating’ compared to ‘foliar spray’ technique. In addition, ‘seed coating’ with a bacterial formulation might deliver higher bacterial concentration to the seeds and consequently to the rhizosphere, in comparison with other methods [17].

## 4. Materials and Methods

### 4.1. Vegetal Material

Three modern durum wheat cultivars (*Triticum durum* Desf.), namely ‘Karim’, ‘Maali’, and ‘Salim’ were used in this study. ‘Karim’ (control) and ‘Maali’ were the most cultivated cultivars in Tunisia, while ‘Salim’ is a new cultivar that was marketed for farmers since 2019–2020 cropping season. The main characteristics of the cultivars are presented in Table S5.

### 4.2. Seed Manipulation and Applied Treatments

The seeds of each cultivar were surface-sterilized with 10% sodium hypochlorite solution for 5 min, and washed three times (5 min each) with distilled water. Afterwards, seeds were soaked for the hydro-priming ([control], distilled water) or coated with (i) a BS named ‘Panoramix’ (Koppert Biological Systems, Rotterdam, The Netherlands), (ii) a chemical product, ‘Spectro’ or ‘Mycoseeds’, or (iii) the combination of two products ‘Panoramix’ and ‘Spectro’ (Table 5). The seeds were placed in gripseal bag and allowed to air before sealing the bag. Then, the bags were shaken to spread the coating material as evenly as possible over the seeds, approximately 30 s to 1 min, and continuous aeration was provided to avoid anxious conditions. For germination bioassay, three BS concentrations (2, 4, and 6 mL kg^−1^ seeds) were tested in order to discern the most effective concentration (Table 5). For the combined treatment, ‘Panoramix + Spectro’, the recommended concentrations were used for both products (i.e., 4 mL kg^−1^ for ‘Panoramix’, see Table 5 for ‘Spectro’). However, based on germination results, the concentration of 2 mL kg^−1^ was used for the individual or combined effect of ‘Panoramix’ for the following trials.

### 4.3. Effectiveness of Biostimulant vs. Chemical Products Using Seed Coating Technique

#### 4.3.1. Seed Germination Bioassay

In this experiment, seven seed coating treatments were considered: Control (distilled water), ‘Panoramix’ (2, 4 and 6 mL kg^−1^ seeds), ‘Spectro’, ‘Mycoseeds’, and ‘Panoramix (4 mL kg^−1^ seeds) + Spectro’. Thereafter, ten coated/non-coated seeds were placed on sterile filter paper (12–15 μm, sterilized at 120 °C for 1 h) in a 90 mm diameter Petri dish moistened with 4 mL of distilled water. Seeds were germinated in dark growth chamber, at 50% relative humidity and an average day/night temperature of 22 ± 2 °C. The completely randomized block design with three replications (n = 3) was adopted, to accommodate the two-way factorial experiment with seven seed coating treatments and three durum wheat cultivars as main factors.

#### 4.3.2. Pot and Field Experiments under Semi-Arid Conditions

Pot and field trials were conducted during 2016/17 cropping season at Boulifa/Kef region (36°7.998′ N 08°42′ E, at 518 m), located in the northwest of Tunisia. The growing season temperature and precipitation data were recorded at the meteorological station of the Kef region. The experimental area has a semi-arid climate with a mean temperature and precipitation varied between 6.65 and 28.35 °C, and 2 to 51 mm from October 2016 to July 2017 (Table S6). The soil of the experimental station is classified as sandy-loam [68] with 98.00 ppm N, 16.53 ppm P, 510 ppm K, and 1.41% of organic matter. The soil of the same station was collected (0–20 cm depth) and used as substrate for pot trial.

The pot trial was conducted in plastic pots (21 cm diameter × 25 cm depth) filled with 5 kg of substrate and placed outdoors. For each pot, ten coated/non-coated seeds were sown on 7 December 2016 thinned to five plants after emergence. Based on germination results, 2 mL kg^−1^ seeds was chosen as concentration for ‘Panoramix’. Thus, the experimental layout was a randomized complete block design with three replicates (n = 3) that included five seed coating treatments (i.e., Control [distilled water], ‘Panoramix’ [2 mL kg^−1^ seeds], ‘Spectro’, ‘Mycoseeds’, and ‘Panoramix [2 mL kg^−1^ seeds] + Spectro’) and three durum wheat cultivars. Supplemental irrigation was applied once a week.

For field trial, three blocs (145 m^2^) subdivided each into 15 plots of 9 m^2^ (in total 45 plots) containing 6 rows of 6 m length with 0.2 m inter-row spacing and 0.5 m inter-plot spacing. Seeds were hand sown on December 10, 2016 at a rate of 350 seeds per m^2^. As described for pot trial, the adopted model of the field experiment was the randomized complete block design with three replicates (n = 3) per treatment (i.e., five seed coating treatments and three cultivars). Before sowing, a basal fertilization of 100 kg ha^−1^ of Di-Ammonium Phosphate (DAP) was applied. Then, a nitrogen (N) fertilization was provided using Ammonium Nitrate (33.5% N), split into three doses of 100 kg ha^−1^ each and applied at 3-leaf (Z13), at tillering (Z26), and at heading (Z32) durum wheat growth stages [69]. Weeds were mechanically controlled using a pre-emergence herbicide, Puma^®^ evolution (fenoxaprop-p-ethyl + iodosulfuron-methyl sodium + mefenpyr-diethyl; Bayer CropScience, Beja, Tunisia) at a rate of 1 L ha^−1^ at the 2–3 leaf stage (Z12-13) [69].

### 4.4. Effectiveness of Biostimulant Supply Methods under Field Conditions

In order to identify the most efficient BS supply method, a second field trial was conducted during 2016/17 cropping season at Boulifa experimental station under the same pedoclimatic conditions. Three methods including (i) ‘seed coating’, (ii) ‘foliar spray’, and (iii) ‘seed coating + foliar spray’ were compared to the control (non-treated). Unit bloc was subdivided into 36 plots of 4.5 m^2^ (3 m × 1.5 m). Each plot was constituted by 6 rows of 3 m length, with a 0.2 m inter-row spacing and a 0.5 m inter-plot spacing. Durum wheat cultivars were sown on 11 December 2016 and a seeding rate of 350 viable seeds per m^2^ was adopted. The seeds were coated only with 2 mL kg^−1^ of ‘Panoramix’, while the foliar spray was applied at the end tillering growth stage (Z29) [69] using 2 mL L^−1^ of ‘Panoramix’. The experiment was arranged with a randomized complete block design using three replicates (n = 3) per treatment (i.e., three BS application methods and three cultivars). The technical operations (i.e., fertilization, weed control) were performed as described above.

### 4.5. Observations and Measurements

For germination bioassay, radicle emergence (>2 mm) was used to determine successful seed germination. The coleoptile (CL, cm) and radicle length (RL, cm) were measured after 4 days, while germination rate (GR, %) was recorded after 7 days.

For pot experiment, three physiological traits were measured, including relative water content, leaf chlorophyll content, and leaf area. At stem elongation stage (Z39) [69], the relative water content (RWC, %) was determined on five plants per pot and calculated according to Clark and Mac Caig [70] using the following formula:

RWC (%) = ((FW−DW)/(TW−DW)) × 100(1)

Variables include FW: fresh weight of harvested leaves; TW: weight of soaked leaves in distilled water for 4 h at room temperature; DW: weight of dried leaves at 80 °C for 24 h.

The relative chlorophyll content (Chl, SPAD) of leaves was estimated using a nondestructive dual-wavelength chlorophyll meter (SPAD-502, Minolta, Japan). The ‘SPAD value’ was determined on flag leaves of five plants per replicate at heading stage (Z59) [69]. The leaf area (LA, cm^2^) was measured also on flag leaves of five plants per pot at Z59 using an electronic planimetre (AM300, Soil Mesures, France).

For pot and field experiments, yield and its related attributes were measured on five plants per pot or per linear meter, including plant height (PH, cm), biomass (B plant^−1^, g), seed number per spike (Seed N S^−1^), thousand kernel weight (TKW, g), and grain yield (GY plant^−1^, g).

To discern the best BS supply method, ten variables were recorded. Chlorophyll content (Chl), plant height (PH, cm), spikelet number per spike (SpkN S^−1^), seed number per spike (Seed N S^−1^), seed weight per spike (Seed W S^−1^, g), and thousand kernel weight (TKW, g) were determined on five plants per m^−2^. However, biomass (B m^−2^, g), spike number (SN m^−2^), and grain yield (GY m^−2^, g) were measured for each plot.

### 4.6. Statistical Data Analysis

When data followed a normal distribution, two-way analysis of variance (ANOVA) was used to evaluate the effect of seed coating treatments (or BS application methods), durum wheat cultivars, and their interaction. The resulting variations in data are expressed as the mean ± standard error (SE) for n = 3. Duncan’s multiple range test at 5% significance level was employed to compare means of seed coating treatments (or BS application methods) and cultivars. All data were analyzed using SPSS software ver. 16.0 (IBM SPSS Statistics. SPSS for Windows, Version 16.0. SPSS Inc., Chicago, IL, USA, 2007). Otherwise, to describe the relationship between ‘treatment-cultivar’ combinations, the principal component analysis (PCA) was performed using R statistical software version 4.0 (The R Foundation for Statistical Computing).

## 5. Conclusions

The current study highlighted the BS, ‘Panoramix’, as an effective and efficient approach in enhancing durum wheat germination using 2 mL kg^−1^ seeds. At this stage, the BS and chemical products acted differently on the above- and below-ground parts of the seedlings; i.e., ‘Panoramix’ enhanced more the root development and inversely for the chemical treatments. Under semi-arid environment, ‘Panoramix’ seems to act by modifying physiological processes in plants to stimulate growth and thereby promote yield and its components. In particular, grain yield and biomass were the most enhanced traits. Considering the durum wheat cultivars, ‘Salim’ showed the best performance using ‘Panoramix’, but in some cases ‘Karim’ valorized better than this BS. Overall, ‘Panoramix’ application by ‘seed coating + foliar spray’ can be efficiently used to enhance growth. Such results ultimately help farmers to develop more profit-oriented behaviors, which are necessary to increase resilience to environmental stress conditions for sustainable durum wheat production.

## Figures and Tables

**Table 1:** Seed coating treatment and cultivar effects on germination attributes. For the three studied variables, the two-way ANOVA is shown according to the different factors.

Sources of Variation	df ^1^	GR (%)	CL (cm)	RL (cm)
Treatment (T)	6	5.63 *** ^2^	25.13 ***	14.66 ***
Cultivar (C)	2	3.06 *	3.820 *	5.66 *
T × C interaction	12	1.26 ^ns^	1.69 ^ns^	2.24 *
Treatments				
Control		79.11b ^3^	6.13a	8.10c
Panoramix (2 mL kg^−1^)		93.33a (15%)	6.91a (11%)	11.64a (30%)
Panoramix (4 mL kg^−1^)		88.88ab (11%)	6.59a (7%)	10.92ab (26%)
Panoramix (6 mL kg^−1^)		91.22a (13%)	6.67a (8%)	10.93ab (26%)
Spectro		81.11b (2%)	4.64b (24%)	8.76c (7%)
Mycoseeds		77.89b (−1%)	3.97c (35%)	8.62c (6%)
Panoramix (4 mL kg^−1^) + Spectro		79.09b (0.1%)	6.76a (9%)	10.06b (19%)
Cultivars				
Karim		78.66b	6.20b	9.04c
Maali		86.84a	7.48a	12.28a
Salim		87.61a	7.15a	11.70b

^1^ df: freedom degree; GR: germination rate; CL: coleoptile length; RL: radicle length. ^2^ ns: non significant; * and ***: significant at 5% and 0.1%, respectively. ^3^ Means with similar letter(s) is not significantly different at 5% probability level according to Duncan’s multiple range test.

**Table 2:** Seed coating treatment and cultivar effects on physiological and agro-morphological attributes under pot conditions. For the eight studied variables, the two-way ANOVA is shown according to the different factors.

Sources of Variation	df ^1^	RWC (%)	Chl (SPAD Values)	LA (cm^2^)	PH (cm)	B Plant^−1^ (g)	Seed N S^−1^	TKW (g)	GY Plant^−1^ (g)
Treatment (T)	4	3.45 * ^2^	3.19 *	21.69 ***	1.13 ^ns^	8.93 *	3.22 *	3.21 *	4.93 **
Cultivar (C)	2	3.52 *	3.78 *	6.28 *	3.57 ^ns^	7.46 *	6.11 *	3.31 *	2.83 *
T × C interaction	8	0.62 ^ns^	2.18 *	2.79 *	3.84 **	2.03 *	2.48 *	1.48 ^ns^	1.07 ^ns^
Treatments									
Control		55.81c ^3^	47.74b	18.48b	55.22a	51.42c	13.89b	33.44c	14.64d
Panoramix (2 mL kg^−1^)		74.95a (25%)	54.79a (13%)	21.51a (14%)	54.56a (−1%)	73.36a (30%)	16.33a (15%)	41.19a (19%)	19.87a (26%)
Spectro		56.99c (2%)	51.08ab (6%)	20.87a (11%)	52.00a (−6%)	54.89c (6%)	14.11b (2%)	35.77b (6%)	17.38ab (16%)
Mycoseeds		58.62ab (5%)	47.90b (0.5%)	19.46ab (5%)	55.00a (−0.4%)	54.80c (6%)	14.33b (3%)	37.72ab (11%)	16.13cd (9%)
Panoramix (2 mL kg^−1^) + Spectro		63.85ab (13%)	48.86b (2%)	20.90a (12%)	52.11a (6%)	65.88b (22%)	15.22ab (9%)	39.80a (16%)	18.53ab (21%)
Cultivars									
Karim		54.33b	46.07b	19.42a	56.33a	52.05c	14.99b	33.30b	14.99b
Maali		66.18a	52.77a	21.85a	55.93a	67.00a	18.88a	38.74a	18.88a
Salim		65.62a	51.38a	20.27ab	55.07a	61.15b	18.07a	39.30a	18.07a

^1^ df: degree of freedom; RWC: relative water content; Chl: chlorophyll content; LA: leaf area; PH: plant height; B plant^−1^: biomass per plant; Seed N S^−1^: seed number per spike, TKW: thousand kernel weight; GY plant^−1^: grain yield per plant. ^2^ ns: non significatif; *, **, and ***: significant at 5%, 1%, and 0.1% respectively. ^3^ Means with similar letter(s) is not significantly different at 5% probability level according to Duncan’s multiple range test.

**Table 3:** Seed coating treatment and cultivar effects on agro-morphological attributes under field conditions. For the five studied variables, the two-way ANOVA is shown according to the different factors.

Sources of Variation	df ^1^	PH (cm)	B Plant^−1^ (g)	Seed N S^−1^	TKW (g)	GY m^−1^ (g)
Treatment (T)	4	2.49 * ^2^	32.21 ***	2.60 ^ns^	20.49 ***	10.89 ***
Cultivar (C)	2	0.39 ^ns^	6.10 **	2.37 *	0.37 ^ns^	4.30 *
T × C interaction	8	0.44 ^ns^	9.66 ***	8.92 ***	8.04 ***	4.46 ***
Treatments						
Control		52.67c ^3^	129.16a	20.06a	36.05b	36.41c
Panoramix (2 mL kg^−1^)		55.44a (5%)	147.59a (12%)	23.28a (14%)	38.17a (5%)	49.58a (27%)
Spectro		54.78ab (4%)	129.09a (0.1%)	19.94a (1%)	36.36b (1%)	40.08bc (9%)
Mycoseeds		52.56c (0.2%)	129.81a (0.5%)	22.72a (12%)	36.39b (1%)	43.02b (15%)
Panoramix (2 mL kg^−1^) + Spectro		55.67a (5%)	145.96a (11%)	20.89a (4%)	37.38ab (4%)	48.31a (25%)
Cultivars						
Karim		52.67a	121.18c	21.80ab	35.12a	40.30b
Maali		54.60a	136.52b	22.67a	37.66a	43.72ab
Salim		54.20a	148.08a	19.67b	38.04a	45.03a

^1^ df: degree of freedom; PH: plant height; B plant^−1^: biomass per plant; Seed N S^−1^: seed number per spike; TKW: thousand weight kernel; GY m^−1^: grain yield per linear meter. ^2^ ns: non significatif; *, **, and ***: significant at 5%, 1%, and 0.1%, respectively. ^3^ Means with similar letter(s) is not significantly different at 5% probability level according to Duncan’s multiple range test.

**Table 4:** Biostimulant supply method and cultivar effects on physiological and agro-morphological attributes under field conditions. For the nine studied variables, the two-way ANOVA is shown according to the different factors.

Sources of Variation	df ^1^	Chl (SPAD Values)	PH (cm)	B m^−2^ (g)	SN m^−2^	Spk N S^−1^	Seed N S^−1^	Seed W S^−1^ (g)	TKW (g)	GY m^−2^ (g)
Treatment	3	6.96 *** ^2^	0.98 ^ns^	8.87 ***	111.14 **	0.25 ^ns^	0.55 ^ns^	5.12 **	1.34 *	73.59 ***
Cultivar	2	0.49 ^ns^	3.08 ^ns^	5.61 ***	1.42 ^ns^	3.77 *	0.46 ^ns^	1.40 **	0.53 *	0.08 **
T × C interaction	6	0.42 ^ns^	1.48 ^ns^	10.40 ***	0.68 ^ns^	1.37 ^ns^	1.45 ^ns^	1.76 ^ns^	1.06 ^ns^	2.92 *
Treatments										
Control		48.58a ^3^	66.19a	1430b	283.00c	12.52b	18.48a	6.03b	39.83bc	252 310b
Seed coating		51.96ab (6%)	64.00a (3%)	1600a (11%)	302.41b (6%)	15.62a (20%)	18.48a (0%)	8.38a (28%)	44.20ab (10%)	325 380a (22%)
Foliar spray		51.44ab (6%)	66.44a (0.4%)	1490b (4%)	282.15c (0.3%)	13.75b (9%)	17.93a (3%)	7.56ab (20%)	42.67bc (7%)	314 780a (20%)
Seed coating + Foliar spray		53.62a (9%)	64.11a (3%)	1610a (11%)	315.19a (10%)	15.47a (19%)	16.59a (10%)	8.94a (32%)	44.40a (10%)	331 920a (24%)
Cultivars										
Karim		48.83b	66.39a	1400b	294.78b	12.77b	17.61b	6.57b	40.61b	291 690b
Maali		52.44a	66.31a	1550ab	297.50a	15.07a	17.33b	8.09a	43.66a	312 030a
Salim		52.93a	62.86a	1660a	294.78b	15.17a	18.67a	8.26a	44.06a	314 570a

^1^ df: freedom degree; Chl: chlorophyll content; PH: plant height; B m^−2^: biomass per m^−2^; SN m^−2^: spike number per m^−2^; SpkN S^−1^: spikelet number per spike; Seed N S^−1^: seed number per spike; Seed W S^−1^: seed weight per spike; TKW: thousand kernel weight; GY m^−2^: grain yield per m^2^. ^2^ ns: non significatif; *, **, and ***: significant at 5%, 1%, and 0.1%, respectively. ^3^ Means with similar letter(s) is not significantly different at 5% probability level according to Duncan’s multiple range test.

**Table 5:** Composition and quantities of products used.

Products	Composition	Quantities
Panoramix	Mix of *Bacillus* spp. (2 × 10^7^ CFU mL^−1^), *Trichoderma* spp. (>1 × 10^7^ CFU mL^−1^), *endomycorrhiza* (>10 propagules mL^−1^) and additives	2 mL per kg of seeds 4 mL per kg of seeds 6 mL per kg of seeds
Mycoseeds FS 60	60 g L^−1^ Tebuconazole	0.5 mL of product + 5 mL of water per kg of seeds
Spectro extreme 115 FS	92 g L^−1^ Difenoconazole + 23 g L^−1^ Métalaxyl-M	0.65 mL of product + 5 mL of water per kg of seeds

## Data Availability

Available upon reasonable request.

## Supplementary Materials

The following are available online at https://www.mdpi.com/article/10.3390/plants11010133/s1: Table S1—Correlation of the seed germination traits with the first two discriminant axes of PCA. Table S2—Correlation of the physiological and agro-morphological traits obtained under pot conditions with the first two discriminant axes of PCA. Table S3—Correlation of the agro-morphological traits obtained under field conditions with the first two discriminant axes of PCA. Table S4—Correlation of the physiological and agro-morphological traits obtained under field conditions with the first two discriminant axes of PCA. Table S5—Main characteristic of used durum wheat cultivars. Table S6—Mean precipitation (mm) and temperature (°C) in Boulifa/Kef site during 2016/17 cropping season.

## Abbreviations

BS	biostimulant
PGPR	plant growth-promoting rhizobacteria
PGPF	plant growth-promoting fungi
AM fungi	arbuscular mycorrhizal fungi
GR	germination rate
CL	coleoptile length
RL	radicle length
RWC	relative water content
Chl	chlorophyll content
LA	leaf area
PH	plant height
B	Biomass
SN	spike number
SpkN S^−1^	spikelet number per spike
Seed N S^−1^	seed number per spike
Seed W S^−1^	seed weight per spike
TKW	thousand kernel weight
GY	grain yield
