# Supplementary material for: Use of Biostimulants: Towards Sustainable Approach to Enhance Durum Wheat Performances
Source: Plants (Basel). 2022 Jan 4;11(1):133. doi: 10.3390/plants11010133 (PMC8747104; doi:10.3390/plants11010133)
Supplement: Supplementary file 1 [file plants-11-00133-s001.zip › plants-1497820-supplementary.pdf]

Supplementary Materials

Table S1. Correlation of the seed germination traits with the first two discriminant axes of PCA.

Traits	PC1	PC2
RL	0.601	-0.260
CL	0.544	0.825
GR	0.585	-0.502

GR: germination rate; CL: coleoptile length; RL: radicle length.

Table S2. Correlation of the physiological and agro-morphological traits obtained under pot conditions with the first two discriminant axes of PCA.

Traits	PC1	PC2
Seed N S^{-1}	0.363	0.561
LA	0.368	-0.656
B $plant^{-1}$	0.383	-0.162
GY $plant^{-1}$	0.389	-0.204
TKW	0.387	0.232
Chl	0.373	-0.113
RWC	0.381	0.347

RWC: relative water content; Chl: chlorophyll content; LA: leaf area; B $plant^{-1}$: biomass per plant; Seed N S^{-1}: seed number per spike, TKW: thousand kernel weight; GY $plant^{-1}$: grain yield per plant.

Table S3. Correlation of the agro-morphological traits obtained under field conditions with the first two discriminant axes of PCA.

Traits	PC1	PC2
GY $plant^{-1}$	0.504	-0.355
B $plant^{-1}$	0.524	-0.408
TKW	0.527	0.045
PH	0.440	0.839

PH: plant height; B $plant^{-1}$: biomass per plant; TKW: thousand weight kernel; GY $plant^{-1}$: grain yield per plant.

Table S4. Correlation of the physiological and agro-morphological traits obtained under field conditions with the first two discriminant axes of PCA.

Traits	PC1	PC2
Seed WS^{-1}	0.447	-0.070
GY plot^{-1}	0.403	-0.197
TKW	0.434	0.236
Chl	0.433	0.193
B plot^{-1}	0.393	0.474
SN plot^{-1}	0.327	-0.800

Chl: chlorophyll content; B plot^{-1}: biomass per plot; SN plot^{-1}: spike number per plot; Seed W S^{-1}: seed weight per spike; TKW: thousand kernel weight; GY plot^{-1}: grain yield per plot.

Table S5. Main characteristic of used durum wheat cultivars.

Name	Origin	Main characteristics
Karim	Cross made at CIMMYT/Mexico and released in 1980	Semi-early cultivar<br>Productive cultivar<br>Susceptible to leaf rust and septoria diseases<br>Specific weight: 78-82 kg hl^{-1}<br>Thousand kernel weight: 40-45 g<br>Proteins: 11-15%
Maali	Local crossing and released in 2007	Early cultivar<br>Very productive (25% more than Karim)<br>Resistant to powdery mildew, and moderately resistant to septoria and leaf rust<br>Tolerant to drought<br>Specific weight: 82-84 kg hl^{-1}<br>Thousand kernel weight: 40-45 g<br>Proteins: 12-15%
Salim	Local crossing and released in 2009	Early cultivar<br>Very productive cultivar<br>Resistant to septoria, leaf rust, and to yellow rust<br>Tolerant to drought<br>Specific weight: 80-83 kg hl^{-1}<br>Thousand kernel weight: 40-48 g<br>Proteins: 12-13.5%

Table S6. Mean precipitation (mm) and temperature (°C) in Boulifa/Kef site during 2016/17 cropping season.

Meteological data	Oct	Nov	Dec	Jan	Feb	Mar	Apr	May	Jun	Jul
Precipitation (mm)	11	43	51	32	24	2	40	2	37	15
Temperature (°C)	20.75	14.3	10.05	6.65	12.5	12.3	16.95	21.15	26.5	28.35
